# Hydrogen Sensors Based on Pd-Based Materials: A Review

**DOI:** 10.3390/s25113402

**Published:** 2025-05-28

**Authors:** Shubin Yan, Yuhao Cao, Yiru Su, Biyi Huang, Changxin Chen, Xianfeng Yu, Aiwei Xu, Taiquan Wu

**Affiliations:** 1 School of Electrical Engineering, Zhejiang University of Water Resources and Electric Power, Hangzhou 310018, China; caoyuhao512@163.com (Y.C.); suyr@zjweu.edu.cn (Y.S.); huangby@zjweu.edu.cn (B.H.); yuxf@zjweu.edu.cn (X.Y.); awxu@zjweu.edu.cn (A.X.); tqwu@zjweu.edu.cn (T.W.); 2 Joint Laboratory of Intelligent Equipment and System for Water Conservancy and Hydropower Safety Monitoring of Zhejiang Province and Belarus, Hangzhou 310018, China; 3 School of Electrical and Control Engineering, North University of China, Taiyuan 030051, China; chenchangxin@nuc.edu.cn

**Keywords:** hydrogen sensor, palladium, thin film, testing technology, hydrogen-sensitive material

## Abstract

Hydrogen is receiving a lot of attention from researchers as a clean energy source and one of the most promising sources of energy for the future. Detection of hydrogen before it reaches explosive conditions is a central issue in the safe use of hydrogen. Hydrogen sensors are devices that detect the hydrogen concentration in the environment and are capable of outputting an electrical signal proportional to the magnitude of the hydrogen concentration. Palladium (Pd) has become one of the preferred materials for the preparation of hydrogen sensors due to its strong hydrogen absorbing ability. In this paper, the intrinsic mechanism of hydrogen absorption by Pd metal is revealed, and the performance of various types of Pd-based hydrogen sensors is reviewed.

## 1. Introduction

Hydrogen is a clean gas without color and odor, which has the potential to be one of the important solutions to the problem of the world’s energy reserves, global warming, and carbon emissions [1,2,3]. Compared with the other flammable gases, such as methane and gasoline, hydrogen has many characterizations, including extremely low density ($0.899\;\mathrm{kg}/m^{3}$), high combustion calories (141 kJ/g) and high diffusion coefficient ($0.61\;{\mathrm{cm}}^{2}/s$ in the air). Due to hydrogen’s strong oxidation, it reacts chemically with many metals. This makes hydrogen require special protection during transportation and use [4,5,6,7]. The security of hydrogen use is the extremely important thing in the large-scale commercialization and also the urgent issue to be resolved. Nevertheless, accurate and fast sensing technology and equipment are important prerequisites for the safe use of hydrogen [8,9].

A hydrogen sensor is a detection apparatus that transforms hydrogen concentration into an electrical signal [10,11]. Compared with the conventional hydrogen testing methods (such as the gas chromatography method and oxidation reduction potential (ORP) tester), the hydrogen sensor has many advantages, including smaller size, faster response time, and the capability of real-time monitoring. With the booming development of low-carbon manufacturing and hydrogen energy emerging industries, the demand for high-performance hydrogen sensors continues to expand, and researchers are committed to the development of a hydrogen sensor with high sensitivity, short response time, high stability, small size, and low cost [12,13,14,15]. The key performance indicators of the developed hydrogen sensor include response percentage, response time, recovery time, and gas selectivity. High-performance hydrogen sensors are vital for ensuring safe production, environmental protection, and energy security and play a crucial role in various applications [16,17,18].

**Figure 1:**
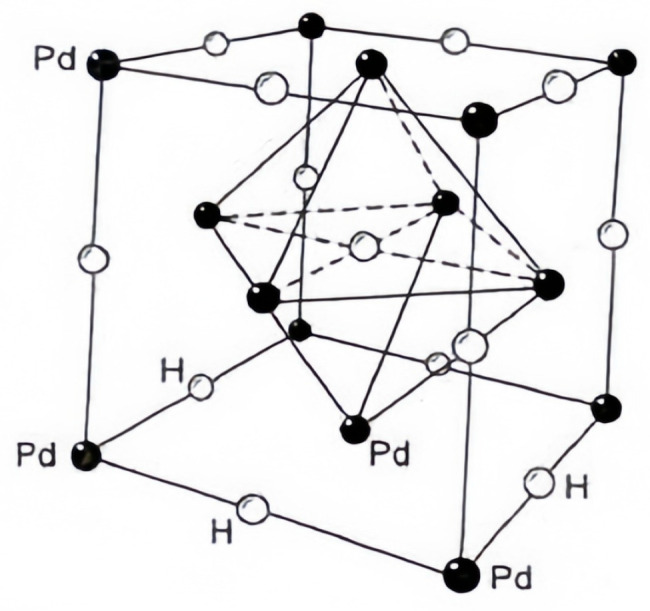
Schematic diagram of palladium hydrogen compound structure.

Previous research efforts explored numerous materials for hydrogen-sensitive sensors. Among these, palladium metal emerged as an optimal choice for fabricating hydrogen sensors, owing to its exceptional sensitivity and stability towards hydrogen [19,20]. Palladium is highly selective for hydrogen and has a high sensitivity, with one volume of palladium absorbing about 886 times its own volume of hydrogen [21,22,23,24]. The principle of interaction between palladium metal and hydrogen molecules can be understood as a dissolution-diffusion model, where the hydrogen molecules adsorb on the surface of the palladium metal and dissociate into hydrogen atoms, which dissolve in the palladium metal to form palladium-hydrogen compounds (${\mathrm{PdH}}_{x}$), as shown in Figure 1. Hydrogen atoms are located in the interstices of the palladium metal lattice and are free to move around. Driven by the external concentration of hydrogen, the hydrogen atoms inside the palladium metal will diffuse outside the palladium metal and recombine to form hydrogen molecules for detachment [25,26,27,28,29,30].

This paper describes the working principles of different types of palladium-based hydrogen sensors based on existing hydrogen sensing technologies and reviews the structural and material improvements of various types of sensors, as well as summarizes and looks forward to the prospects of future hydrogen sensor applications [31].

## 2. Principle of Detection

In order to better prepare Pd-based hydrogen sensors, a better understanding of the mechanisms by which hydrogen moves in and out of palladium metal is needed [32]. The Pd and hydrogen-specific reaction process is as follows: when the Pd metal is placed in the hydrogen environment, H_2_ molecules will be adsorbed on the surface of the film and decomposed into H atoms, H atoms penetrate into the interstitial sites of the Pd lattice, resulting in the formation of ${\mathrm{PdH}}_{x}$ compounds. When the concentration of H_2_ in the environment decreases, H atoms will desorb and dissociate from Pd atoms [33]. Therefore, the Pd-H reaction is a reversible reaction; the reaction equation is shown below:

(1) $$Pd+\frac{x}{2}H_{2}↔{PdH}_{x}$$

When the hydrogen concentration in the environment is low, α-phase ${\mathrm{PdH}}_{x}$ will be generated, this reaction is irreversible, if the number of permeated H atoms exceeds the upper limit of maintaining the α-phase, the volume expansion will occur after the absorption of H by the Pd metal, and the pure Pd film will easily generate excessive α-phase ${\mathrm{PdH}}_{x}$, which will cause irrecoverable volume expansion and even blistering, rupture, and other “hydrogen embrittlement” phenomena, which will damage the sensor’s hydrogen-sensitive materials and impair the reproducibility of the sensor [34,35]. When the hydrogen concentration in the environment is high, β-phase ${\mathrm{PdH}}_{x}$ is formed, and this reaction is reversible. In order to produce more β-phase ${\mathrm{PdH}}_{x}$, other metal materials are usually doped into the Pd-based hydrogen-sensitive materials to lower their lattice constants, preventing H atoms from penetrating into the lattice gap of the Pd-based materials. This inhibits the “hydrogen embrittlement phenomenon” and improves the stability and repeatability of the sensor [36].

The entry of H atoms into the Pd metal lattice leads to a decrease in the number of free electrons per unit volume n in the Pd metal lattice and a decrease in the electron mean free range, according to the resistivity microscopic equation [37]:

(2) $$\rho =\frac{2mv}{ne^{2}L}$$

where ρ is the resistivity of the Pd metal, e is the electron charge, v is the electron rate, m is the electron mass, and L is the electron mean free range.

This shows that the entry of H atoms into the Pd metal increases the resistance. The resistance of Pd metal rises by an order of magnitude proportional to the number of hydrogen atoms absorbed by the Pd metal, which is mathematically expressed as follows according to Sieverts’ law [38]:

(3) $$\left[\%H\right]=K\sqrt{\frac{pH_{2}}{p\theta }}$$

where K represents the Sieverts constant, which is numerically equal to the solubility of hydrogen when the partial pressure of hydrogen is 101.3 kPa at a certain temperature. [%H] represents the solubility of hydrogen in palladium metal, $\rho H_{2}$ is the partial pressure of hydrogen, and ρθ is the standard atmospheric pressure.

It should be pointed out that in Equation (3), the resistance is numerically not equal to the solubility of hydrogen. The changes in resistance, responsiveness, etc., relative to the reference values are only proportional to the square root of the relative hydrogen concentration (i.e., hydrogen partial pressure) [39,40].

## 3. Palladium-Based Hydrogen Sensitive Materials Hydrogen Sensors

### 3.1. Catalytic Hydrogen Sensor

**Figure 2:**
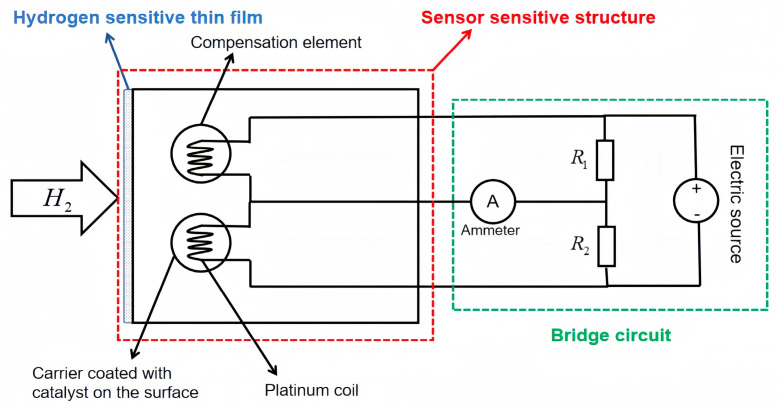
Schematic diagram of catalytic hydrogen sensor structure and principle.

Catalytic sensors work on the principle that combustible gases react with oxygen on the surface of the sensor to generate heat, and the heat generated by the catalytic combustion of the combustible gases is transferred to the platinum coils, causing the resistance of the platinum coils to increase [41]. Various electronic components are used to form a bridge, the coil resistance increases to cause voltage changes in the bridge circuit, and the voltage increase is proportional to the change in gas concentration, as shown in Figure 2. Many combustible gas sensors, including hydrogen, can be prepared using this principle [42].

Catalytic sensors feature a long lifespan, high precision, and rapid response times. However, their notable limitation lies in low gas selectivity. In recent years, researchers have conducted extensive studies aimed at downsizing these sensors and enhancing catalyst selectivity. Ivanov et al. [43] investigated several platinum group catalysts to quantitatively compare the response and sensitivity of hydrogen sensors to hydrogen at the lower explosive limit. It was found that the use of metallic iridium (Ir) and metallic rhodium (Rh) as catalysts can effectively improve the low-temperature performance of the hydrogen sensor with Pd as a catalyst. The catalyst has been experimented with to achieve the best detection effect above 400, which can be accurately detected in the concentration range before the explosion. However, the principle of operation of catalytic hydrogen sensors is very different from the principle of hydrogen adsorption by palladium metal, so catalytic hydrogen sensors are rarely available with palladium metal as the sensitive material.

Catalytic hydrogen sensors offer the advantages of high detection accuracy, fast response time, and long service life. However, these sensors will burn and generate a lot of heat during operation, which may cause an explosion, and are therefore not suitable for use in confined spaces.

### 3.2. Current Hydrogen Sensors

**Figure 3:**
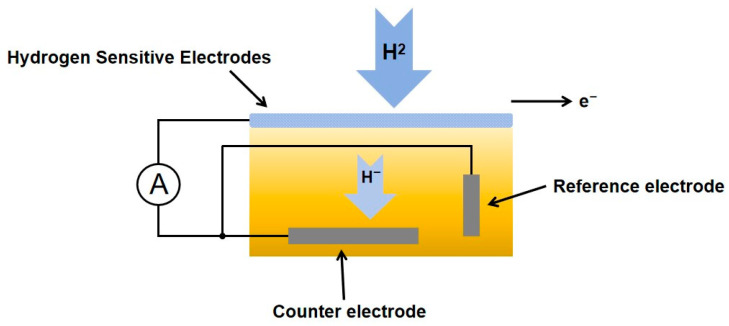
Schematic structure of the current hydrogen sensor.

The detection principle of current-based hydrogen sensors is based on a change in current caused by a chemical reaction between hydrogen and the surface of the hydrogen-sensitive material, as shown in Figure 3. When hydrogen enters the sensor, hydrogen atoms react chemically with the surface of the sensor material, resulting in a change in the electrical properties of the material. This chemical reaction involves the hydrogen atoms interacting with elements such as oxygen and nitrogen on the surface of the material, which changes the electrical conductivity of the material. The change in the electrical properties of the hydrogen-sensitive material affects the charge distribution and carrier concentration within the material [44]. This charge transfer or redistribution can lead to changes in the current in the sensor. The change in current in the sensor can be detected and measured in real time by an external circuit. The circuitry established between the electrodes of the sensor is affected by changes in the electrical properties of the material, thus reflecting changes in hydrogen concentration.

In recent years, current-based hydrogen sensors prepared with Pd-based sensitive materials as sensitive materials have had good signal detection and smaller current signal drift. Kadhim et al. [45] prepared a metal-semiconductor-metal hydrogen sensor using nanocrystalline ${\mathrm{SnO}}_{2}$ and Pd metal, with glycerol added to the ${\mathrm{SnO}}_{2}$ film grown using the gel-sol method to form Schottky contacts. The hydrogen sensor it prepared has a detection sensitivity of 95% for 1000 ppm hydrogen ambient at room temperature and up to 160% at 125 °C. Lee et al. [46] prepared alloyed Pd-Ni alloy films on flexible PDMS substrates to reduce the nanogap to less than 100 nm, which can open or close the nanogap during the adsorption of $H_{2}$ to form a highly sensitive hydrogen sensor. The detection lower limit of this sensor can be as low as 0.01%, and the response time at room temperature is 1 s with a recovery time of less than 3 s. Ayesh et al. [47] prepared a hydrogen sensor based on Pd-Cu alloy nanoclusters by magnetron sputtering and inert gas condensation in an ultra-high vacuum compatible system (UHV). The carriers in the sensor are mainly transported in a metal network dominated by tunneling. The prepared hydrogen sensor is capable of detecting hydrogen concentrations as low as 0.5% in air; in addition, its conductivity varies up to % in a hydrogen concentration of 5%. The main advantage of this sensor is that the hydrogen concentration is linear with the sensitivity, which makes it easy to calibrate. Xiang et al. [48] prepared a hydrogen sensor with Pd nanoparticles doped ${\mathrm{TiO}}_{2}$ into nanotube films as the sensitive material. The incorporation of ${\mathrm{TiO}}_{2}$ nanotubes significantly enhances the performance of Pd nanoparticles due to the presence of Schottky barriers, in which the catalytic effect of the Pd nanoparticles is the key to reducing the operating temperature of the sensor to room temperature. The sensitivity of the sensor was reduced from 11.3% to 8.3% when the temperature was raised from 25 °C to 100 °C, and its response time and recovery time were both 2 min at room temperature. More importantly, the sensor showed good selectivity to $H_{2}$, and ${\mathrm{CO}}_{2}$ and ${\mathrm{CH}}_{4}$ at the same concentration caused almost no change in conductivity. Alenezy et al. [49] prepared a current-based hydrogen sensor based on Pd-modified long-range ordered ${\mathrm{TiO}}_{2}$ crystals as a sensitive material. The use of Pd nanoparticles as a dissociation catalyst and the application of an external stimulus, which increased the reaction rate and electron flow of hydrogen over the catalytically sensitive layer, enabled the detection of $H_{2}$ gases with concentrations as low as 50 ppm in this particular layered structure, and the sensor was extremely selective (>93%) for $H_{2}$. Arora et al. [50] developed a three-electrode electrochemical hydrogen sensor prepared from nanocomposites made of palladium oxide-reduced graphene oxide (PdO-rGO), the nanocomposites displaying a large surface area dedicated to hydrogen adsorption. The sensor exhibited remarkable sensitivity and a consistent linear response to a hydrogen concentration of 0.462 μA/%, while the stability and gas selectivity of the sensor were greatly improved. Lee et al. [51] developed a highly sensitive hydrogen sensor based on palladium nanoparticles (PdNPs)-modified metastasis-free three-dimensional (3D) graphene. 3D graphene provides a larger surface area for gas adsorption and reaction, and the introduction of PdNPs improves the electron transport properties and enhances the sensitivity. The sensor achieved a gas response value of 41.9% in a 3% hydrogen environment.

The most outstanding advantage of current-type hydrogen sensors is the strong stability and fast response speed, but because the measurement signal of current-type hydrogen sensors is a current signal, it requires a special current acquisition device to collect the signal and convert it into a digital signal, which requires a high level of user skills, and at the same time, the environmental parameters will also have a certain impact on the current signal, such as temperature, humidity, pressure, and so on.

### 3.3. Resistive Hydrogen Sensors

**Figure 4:**
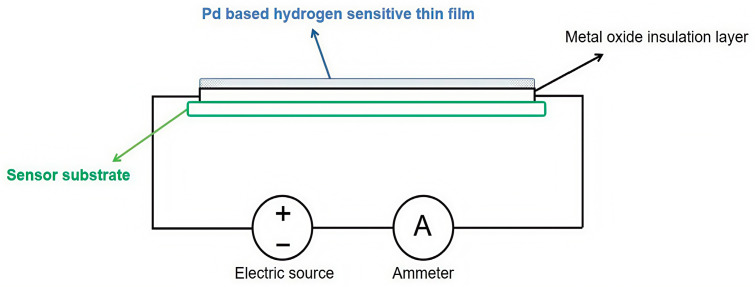
Schematic diagram of resistance-type hydrogen sensor structure and principle.

Resistive hydrogen sensors utilize the principle that exposure of Pd-based materials to H_2_ will first cause a change in the resistivity of the material to detect hydrogen. When the ambient hydrogen concentration changes, the resistance of the metal oxide is measured to obtain the hydrogen concentration, and the relationship is approximately linear [52]. The structure and principle of the resistive hydrogen sensor is shown in Figure 4.

Pd-based resistive hydrogen sensors are mainly based on the adsorption of palladium (Pd) on hydrogen. Hydrogen molecules adsorbed on the surface of Pd diffuse into the interior so that the electronic structure of Pd and lattice parameters change, which leads to changes in the electrical resistance of Pd, and to determine the concentration of hydrogen through the detection of changes in the electrical resistance, which relies on changes in electrical resistance brought about by changes in the structure of the material on the physical layer and electronic structure. The Pd-based current-type hydrogen sensor is based on the chemical reaction between hydrogen and the surface of the Pd-based hydrogen-sensitive materials, hydrogen into the sensor, hydrogen atoms interact with the surface of the material, such as oxygen, nitrogen and other elements, so that the material’s electrical properties change, affecting the material’s internal charge distribution and carrier concentration, which ultimately leads to changes in the current in the sensor, and the current changes through the real-time external circuitry to detect the current changes in order to reflect the concentration of hydrogen, and the core of the process lies in the charge transfer and current change triggered by the electrochemical reaction. The Pd-based resistive type detects resistance changes due to physical changes, while the Pd-based current type detects current changes triggered by electrochemical reactions. There are significant differences between the two in terms of the detection principle, the mechanism of the dependent changes, and the type of the detected signals.

Resistive hydrogen sensors prepared from pure Pd metal materials have the advantages of simple preparation and good performance. Zhang et al. [53] prepared a Pd-based resistive hydrogen sensor by the chemical bath method. They immersed PAN substrate material in a solution containing Pd, and the Pd in the solution underwent a chemical reaction on the substrate surface, thereby forming a Pd film on the PAN substrate surface. Meanwhile, in order to eliminate the influence of zero drift on the performance of the sensor, they carried out a simple annealing treatment on the sensor samples. After testing, the sensor shows excellent stability and high sensitivity with a lower detection limit of 2 ppm, and it is worth mentioning that the sensor shows good mechanical flexibility with a detection difference of less than 0.01% under bending conditions. Hydrogen sensors prepared from pure Pd materials will unavoidably cause hydrogen embrittlement of the material, which affects the performance of the sensor. In general, in order to improve the performance of hydrogen sensors, resistive hydrogen sensors can be improved by surface modification of palladium-based materials or addition of nanoparticles. Nanoscale Pd-based hydrogen-sensitive materials mainly include nanofilms, nanoparticles, nanowires, and bimetallic nanomaterials. Due to the smaller size and surface area of Pd nanostructures, the phase transition of Pd materials can be reduced to improve the performance of the sensors. Hu et al. [54] prepared nanoporous palladium films using a DC sputtering process and annealing. The structures were more strongly crystallized, with larger grain sizes, and had good linear properties for concentrations of hydrogen ranging from 0.004 to 1.2%. Its response time was 25 s, and recovery time was 10 s. Yu et al. [55] used a new chemical plating method to deposit palladium nanotubes within the micropores of polycarbonate membranes. The nanotube films prepared by this method have the advantages of low price and high sensitivity. Nanotube structures have a larger surface area and can generate more adsorption sites. The lower limit of detection is 500 ppm. RaviPrakash et al. [56] prepared a hydrogen sensor with dense columnar Pd nanostructured thin films as the sensitive material using the DC magnetron sputtering technique, which showed good gas selectivity in $H_{2}$ and CO gas environments and was tested with a lower limit of detection of 1% and a response time of 500 s. Lundström et al. [57] prepared a Pd-based hydrogen sensor with a grating structure, which demonstrated good sensitivity with a lower limit of up to 10 ppm and good stability at low hydrogen concentrations. Stiblert et al. [58], similarly, prepared a Pd grid structure hydrogen detection device, which can detect hydrogen at concentrations as low as 1 ppm. Kumar et al. [59] used a colloidal sol-gel synthesis method to fabricate Pd nanowires with a diameter less than 5 nm. The sensor demonstrated good performance for a hydrogen environment with a concentration of 1% and was able to achieve a response time of 3.4 s and a recovery time of 11 s. In addition, the sensor demonstrated good stability and reproducibility. Thokala et al. [60] prepared a novel resistive hydrogen sensor using covalent organic framework (COF) and rapid formation of Pd nanoparticles on the surface of COF by reduction reaction method. The sensor has a high response to a 1% hydrogen environment (67.3%) and can achieve a response time of 5.3 s and a recovery time of 3.5 s.

Hydrogen sensors based on metal oxides with Pd decorations are characterized by low power consumption and low preparation cost. Meanwhile, hydrogen sensors prepared with metal compounds are usually able to adapt to different operating environments, even at high temperatures, because metal oxides can maintain good chemical properties at high temperatures. Rashid et al. [61] prepared a flexible hydrogen sensor based on Pd-doped ZnO nanorods. Well-aligned vertical ZnO nanorod arrays were successfully synthesized on polyimide tape substrate using the aqueous solution method, and Pd nanoparticles of about 10 nm in size were deposited as catalysts on ZnO nanorod arrays by magnetron sputtering. The sensor has a lower detection limit of 0.2 ppm and exhibits good stability at room temperature, and more importantly, due to the use of flexible materials, the sensor performance does not deteriorate significantly when bent at 90°. Kien et al. [62] demonstrated a hydrogen sensor prepared by modifying Pd metal on the surface of ${\mathrm{SnO}}_{2}$, which showed good response to $H_{2}$ gas at an operating temperature of 150 °C, with response time and recovery time >200 s. Ayesh et al. [63] demonstrated a hydrogen sensor based on Pd and ${\mathrm{SnO}}_{2}$ nanoclusters prepared by magnetron sputtering and inert gas condensation in an ultra-high vacuum system. The sensor is capable of detecting $H_{2}$ gas at 0.5% concentration, and its response time does not exceed 10 s at 80 °C. Sokovykh et al. [64] conducted research on a ${\mathrm{Pd}/\mathrm{SnO}}_{2}$-based hydrogen sensor prepared by the sol-gel method, can stably detect $H_{2}$ as low as 40 ppm at 261 °C with a great detection range (2–1089 ppm). Deivasegamani et al. [65] prepared a Pd-doped Ce and ${\mathrm{SnO}}_{2}$ chemoresistive hydrogen sensor based on Pd-doped Ce and ${\mathrm{SnO}}_{2}$ using self-selective coating, which has a lower limit of detection of 50 ppm and achieves the best sensing performance in an operating environment 200 °C with a response time of 300 s. Kaur et al. [66] prepared resistive hydrogen sensors based on Pd-doped ${\mathrm{WO}}_{3}$ nanostructures and reduced graphene oxide (RGO) composites, which showed a significantly enhanced selectivity for low concentration (50 ppm) of $H_{2}$ with response time and recovery time of >50 s at an operating environment of 150 °C. Boudiba et al. [67] prepared a resistive hydrogen sensor based on Pd-doped ${\mathrm{WO}}_{3}$ by a promoted method and annealed it at 500 °C. The sensor is capable of detecting $H_{2}$ at concentrations as low as 200 ppm and has a response time of >10 min at an operating temperature of 200 °C. Subsequently, they prepared hydrogen sensors loaded with Pd and ${\mathrm{WO}}_{3}$ on alumina substrates equipped with Au electrodes by screen-printing [68], which were capable of detecting $H_{2}$ at concentrations of 50–200 ppm and achieved optimal performance at 240 °C, when the response time was 24 s. Duan et al. [69] presented a hydrogen sensor prepared from Pd-SnO_2_/rGO ternary composites with a porous structure, which had the best performance in an $H_{2}$ environment with a concentration of 200 ppm with a response time of 25.8 s. Moreover, the sensor demonstrated a good performance in the detection of very low hydrogen concentrations, with the lowest lower detection limit of 0.5 ppm. Wang et al. [70] used the colloidal crystal template method to prepare a hydrogen sensor with a three-dimensional ordered macroporous (3DOM) structure ${\mathrm{WO}}_{3}$ mixed with Pd as a hydrogen-sensitive material, which was able to accomplish a fast response to 50 ppm $H_{2}$’s with a response time of 10 s and a recovery time of 50 s under the environment of 130 °C. Woo et al. [71] presented a hydrogen sensor prepared from Pd nanocubes and ${\mathrm{TiO}}_{2}$ nanofiber composites, which has good linearity to $H_{2}$ concentration, with a response time of 25 s and a recovery time of up to 1 s for a 0.6% concentration of $H_{2}$ at an operating temperature of 150 °C. Moon et al. [72] prepared Pd-modified ${\mathrm{TiO}}_{2}$ materials with a tubular structure on ${\mathrm{SiO}}_{2}/\mathrm{Si}$ substrates, and the proposed sensors were capable of detecting $H_{2}$ at concentrations as low as 1 ppm with a response time of 30 s and a recovery time of 10 s. Lupan et al. [73] prepared a hydrogen sensor based on Pd-modified ZnO nanowire structure, which has a lower detection limit of up to 1 ppm at room temperature, but at an operating temperature of 200 °C, its detection range becomes narrower and the selectivity is greatly reduced, but the gas response is improved. Vijayalakshmi et al. [74] prepared Pd-modified ZnO nanowire material on an ITO substrate for a hydrogen sensor. Due to the doping of Pd, it makes the crystal gap of the ZnO thin film become smaller, and the average grain size becomes smaller. It has been studied that the best detection is achieved when the doping concentration is 2 at.%, and the lower limit of detection is 100 ppm. Kim et al. [75], modified ZnO nanoparticles using Pd noble metal nanoparticles and enhanced hydrogen sensing performance by UV irradiation. The synthesized material was fully characterized, and the sensing layer was deposited on an electrode-patterned glass substrate to make a transparent sensor, which can detect hydrogen at different temperatures and humidity, and the sensor was tested to have the highest response at 250 °C, and it can also detect a mixture of H_2_ with benzene and toluene. The sensor is highly responsive, selective, and reproducible.

The sensitive material of hydrogen sensors is the key to improving their performance, and in recent years, researchers have shown good performance of resistive hydrogen sensors prepared by doping Pd with other metals. At the same time, the doping of other metals changes the lattice structure of Pd, which can enhance the ability of the hydrogen sensor to capture $H_{2}$ and, at the same time, reduce the hydrogen embrittlement phenomenon produced by H atoms entering the interior of the Pd lattice. In recent years, resistive hydrogen sensors prepared from composites doped with Pd and other metals have emerged, such as Pd/Pt, Pd/Ag, Pd/Cu, Pd/Mg, Pd/Ni, and so on. Phan et al. [76] chemically synthesized hydrogen sensors prepared with palladium/platinum (Pd/Pt) complexes, which were polymer-assisted growth of highly homogeneous Pd nanocubes as the core and a thin layer of Pt loaded on the exterior of the Pd core as the shell using a two-step chemical method. Subsequently, the Pd/Pt core-shell was further modified by a hydrazine reaction on a graphene substrate, and the final Pd/Pt core-shell hybrid material with graphene was obtained. The sensor is characterized by a large response amplitude, with a maximum response of 36% to 1% $H_{2}$ concentration at room temperature and a response time of 3 min. Sharma et al. [77] prepared a hydrogen sensor using a microelectromechanical systems process with a stoichiometry of 77:23 with palladium/silver (Pd/Ag) thin film as the sensitive material, which was shown to be capable of detecting $H_{2}$ with a lower limit of 100 ppm and a response of 14.27% at a $H_{2}$ concentration of 1000 ppm. Subsequently, they prepared graphene-Pd/Ag nanocomposites for hydrogen sensors, and they found that the coupling structure between graphene and Pd/Ag alloys could improve the sensing performance [78], and due to the high electrical conductivity of graphene, the gas response was 16.2%, and the response time was elevated to 56 s in an H_2_ environment with a concentration of 1000 ppm. Hassan et al. [79], prepared a Pd/Mg bimetallic-based hydrogen sensor using an ultrasmall mesh structure. The wire mesh structure provides the device with good stability and bendability, which provides a new option for wearable hydrogen sensors. It was shown that the sensor is capable of adequately detecting 10–40,000 ppm concentration of $H_{2}$ with a response time of up to 6 s at room temperature. Gautam et al. [80] prepared a hydrogen sensor using magnetron sputtering deposition of Pd/Mg/Pd/Mg/Pd bimetallic multilayer thin films. The “synergistic effect” between the interfaces of the bimetallic films enhances the hydrogen absorbing ability of the device, which makes the sensor able to respond quickly, and the response time of the device is 3.5 s at room temperature. Yoshimura et al. [81] investigated the performance of a hydrogen sensor for Pd/MgPd alloys, which is capable of detecting $H_{2}$ at concentrations as low as 40 ppm, but due to the simplicity of the sensor’s structure, the detection area is small. Lee et al. [82] discussed the effect of different Ni contents on the hydrogen sensing properties of PdNi alloy films from a structural point of view. It was found that Ni doping in Pd films can effectively improve the hydrogen sensing performance of the films. In addition, the sensitivity of PdNi alloy films to $H_{2}$ decreases with the increase of Ni content, which is attributed to the fact that the doping of Ni reduces the lattice constant and the interstitial volume, which allows fewer hydrogen atoms to penetrate into PdNi alloys with high content of Ni. The incorporation of Ni effectively suppressed the hydrogen brittleness phenomenon of the sensor in high concentrations of hydrogen and increased the detectable range of hydrogen. The addition of Ni effectively suppresses the hydrogen embrittlement phenomenon of the sensor under high concentrations of hydrogen and increases the detectable range of hydrogen. Jiang et al. [83] prepared a PdNi thin film hydrogen sensor integrated with a Pt temperature measurement thin film resistor using MEMS technology ${\mathrm{Si}}_{3}N_{4}$ the film was deposited on the top of the Pt film for suppressing hydrogen diffusion and reducing the interference to the temperature measurement, and the temperature obtained from the Pt temperature sensor was used to calibrate the output response of the PdNi thin film hydrogen sensor. The sensor is capable of detecting $H_{2}$ at concentrations as low as 0.3 ppm with a response time of 10 min and a recovery time of 25 min. Subsequently, they investigated the effect of annealing temperature on the hydrogen sensing performance of PdNi alloy thin films [84] and showed that although the lower limit of detection of the concentration of the sensor sample was increased after annealing (10 ppm), its output response was improved. The response time was 5 min and the recovery time was 15 min. Huang et al. [85] prepared hydrogen sensors by depositing PdNi thin films on α-${\mathrm{Al}}_{2}O_{3}$ and γ-${\mathrm{Al}}_{2}O_{3}$ substrates using metal organic chemical vapor deposition. They found that the films deposited on α-${\mathrm{Al}}_{2}O_{3}$ substrates are much more sensitive to hydrogen than γ-${\mathrm{Al}}_{2}O_{3}$ substrates due to the porous discontinuity characteristic of the larger pores of the substrates. The lower limit of the concentration detection is 0.7%, and the response time is 60 s. The sensor detects only 2–4% of the concentration of the PdNi film deposited on the γ-${\mathrm{Al}}_{2}O_{3}$ surface, and at 4% concentration, the film is cracked and hydrogen embrittlement occurs. Wang et al. [86], using MEMS technology, prepared a resistive hydrogen sensor capable of detecting ppb-scale concentrations. The sensor utilizes a Wheatstone bridge structure with Pd-Au alloy films sputtered on all four resistive arms. The sensor was tested to have a detection limit as low as 20 ppb with a power consumption of only 4.6 mW, and the response and recovery times of the sensor for 3 *v*/*v*% hydrogen were 6 s and 19 s, respectively.

### 3.4. Fiber Optic Hydrogen Sensors

**Figure 5:**
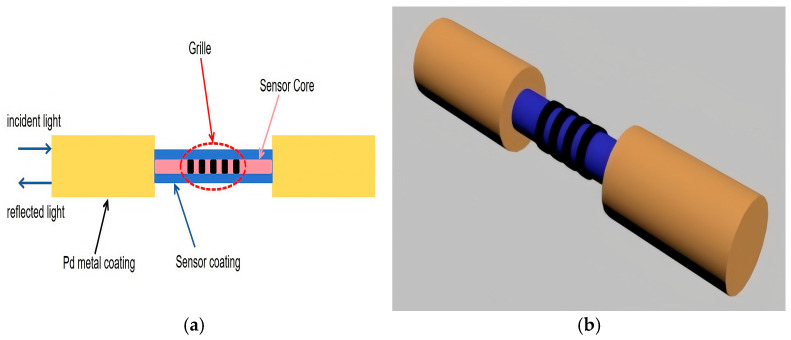
Structure diagram of fiber optic hydrogen sensor. (**a**) Two-dimensional structure diagram; (**b**) Three-dimensional structure diagram.

Currently, most hydrogen sensors are of the electrical or catalytic type, which are based on electrochemical principles. Despite the advantages of simple production and good performance, they are prone to electric sparks during use due to high operating temperatures, which is likely to become a combustion source for igniting high concentrations of hydrogen during the use of the sensor, causing unnecessary casualties and property damage [87,88]. Fiber optic hydrogen sensors are hydrogen sensors prepared on the basis of various optical properties, with unique safety and anti-interference capabilities, and are expected to be the primary choice for the next generation of high-performance hydrogen sensors. Fiber-optic hydrogen sensors use an optical fiber combined with a hydrogen-sensitive material. When the hydrogen-sensitive material reacts with hydrogen, the physical properties of the fiber change, resulting in a change in the optical properties of the transmitted light in the fiber [89]. The hydrogen concentration is measured by detecting the change of the physical quantity corresponding to the output light. The structure is shown in Figure 5. Specifically, when hydrogen undergoes a chemical reaction with PD-based metals to form metal hydrides, it leads to the expansion of Pd metals. This volume change affects the transmission characteristics of optical fibers, such as light loss and phase. By detecting the changes in these optical transmission characteristics, the concentration of hydrogen can be detected. In addition, for the hydrogen sensor with a periodic structure constructed on the optical fiber, when hydrogen enters the Pd metal, the Pd metal expands. This expansion acts on the optical fiber grating, causing changes in physical quantities such as temperature and stress of the optical fiber grating, which in turn affects its optical properties, such as the drift of the central wavelength and the change of reflectivity. The detection of hydrogen can be achieved by detecting the changes in these optical properties. Fiber optic type hydrogen sensors have the advantages of high corrosion resistance and safety [90]. However, the use of Pd metal as the sensitive material also has the effect of hydrogen embrittlement phenomenon on its repeatability.

Fiber optic hydrogen sensors are mainly of the following types, abrupt sensors, micromirror sensors, surface plasmon resonance sensors (SPR), Raman scattering sensors, and fiber Bragg grating sensors (FBG) [91]. Among these, FBG hydrogen sensors are the most common fiber optic hydrogen sensors because they can reduce the effect of light source intensity and thus improve the accuracy of the sensor. FBG refers to the construction of a spatial phase periodically distributed grating in the fiber core; when light passes through the grating, a certain wavelength of light will be reflected. The reflected wavelength is related to the period and the effective refractive index, also known as the Bragg wavelength, which is as follows [92,93,94]:

(4) $$\lambda_{A}=2n_{eff}V$$

In Equation (4), $n_{eff}$ is the effective refractive index of the fiber, and V is the grating period.

Zhou et al. [95] used a femtosecond laser to fabricate a sensor probe with a double-helix microstructure. They set the pitch of a fiber grating sensor with a double-helix structure to 90 μm and sputtered it on a Pd/Ag film, which had a sensitivity of up to 51.5 pm/%H and a response time of 2 min in an $H_{2}$ environment with a concentration of 4%. Subsequently, they tried to coat 520 nm Pd/Ni and Pd/Ag alloy films on a 60 μm a fiber grating with a double helix structure [96] and showed that the fiber grating hydrogen sensor coated with a Pd/Ni film had a sensitivity of 13 pm/%H, while the fiber grating hydrogen sensor coated with a Pd/Ag film had a sensitivity of 25 pm/%H. Coelho et al. [97] investigated a fiber Bragg grating hydrogen sensor clad with Pd metal, using an etching technique to reduce the diameter of the grating to 50 μm, and their design of the grating structure in which one grating is not clad with Pd and one is clad with Pd metal. The sensitivity of the sensor is greater than 20 pm/%H and the response time is 2 min. Dai et al. [98] sputtered a 140 nm Pd/Ni alloy film on a 21 μm a Bragg grating using a thin polypropylene sheet as a protective substrate, which greatly improved the sensitivity of the fiber grating sensor. The sensor has good stability, and its performance deteriorated only after 6 months, but the performance remained good. Fisser et al. [99] sputtered a Pd metal film with a thickness of 20 μm on a fiber Bragg grating and used data processing to compensate for the effects of wavelength instability and room temperature fluctuations on the performance of the sensor, which was shown to have a sensitivity of 96 pm/%H for a concentration of 5% $H_{2}$ at a temperature of 90 °C. Hu et al. [100] developed a π displacement-based fiber Bragg grating hydrogen sensor, which is coated with a Pd/Ta alloy layer to achieve in-situ temperature compensation, and the maximum sensitivity of the sensor after temperature compensation can reach 13.2 pm/%H. Jiang et al. [101] proposed a fiber Bragg grating hydrogen sensor after side polishing and sputtering Pd/Ag alloy film. After the side of the sensor is polished, the residual thickness is 20 μm and a Pd/Ag alloy film with a thickness of 560 nm is sputtered. The sensor is 11.4 times more sensitive than the traditional fiber grating sensor, and its sensitivity can reach 4770 pm/%H. It is worth mentioning that the sensor can be used for the on-line detection of $H_{2}$ gas dissolved in transformer oil with a sensitivity of 2.1 μL/L. Silva et al. [102] prepared a 50 μm diameter conical fiber Bragg grating hydrogen sensor by femtosecond radar technology and coated 150 nm Pd film on the grating. The sensor can accurately detect $H_{2}$ in the concentration range of 0–1%, with a sensitivity of 81.8 pm/%H. Okazaki et al. [103] developed a robust and reliable hydrogen sensor based on fiber Bragg grating (FBG), which utilizes the catalytic heat production of hydrogen over a Pd-loaded silica catalyst to trigger temperature changes for sensing. After optimizing the catalyst preparation, the device produces more heat and has good stability. The fabricated sensor has a lower detection limit of about 0.2% under stable ambient temperature conditions and reliable long-term performance. Yang et al. [104] developed a compact fiber optic sensor with a tilted Bragg fiber grating (TFBG) etched in the fiber core and covered with a 40 nm thick combined film of palladium and tungsten trioxide (Pd/WO_3_). The TFBG-excited cladding resonance, which partially matches the refractive index of the Pd/WO_3_ coating, is highly sensitive to the change of hydrogen concentration. The sensor has excellent performance, with a response time of less than 10 s over the 0–3% concentration range, high repeatability over tens of measurements, and a linear response of over 99.6%, demonstrating good hydrogen sensing characteristics. Dai et al. [105] developed and investigated a novel compact fiber-optic hydrogen sensor based on light intensity demodulation with controlled photothermal technology. The system employs three photodetectors for optical signal conversion, which are used to calibrate and detect the reflected optical signals from a single-mode fiber coated with a WO_3_-Pd_2_Pt-Pt composite film and to receive the reflected optical power from a short fiber Bragg grating whose center wavelength is in the steep wavelength range of the ASE light source. Utilizing a 980 nm laser and a PID controller to operate the hydrogen-sensitive film at 60 °C, the sensing system has a response time of only 0.4 s for 10,000 ppm hydrogen in air and a detection limit of 5 ppm. Zhi et al. [106] proposed and experimented with an optical hydrogen sensor, which utilizes a Bragg grating-functionalized superstructure with a thin palladium film in a helical-core optical fiber for simultaneous detection of hydrogen concentration and temperature based on the cross-sensing of two sets of gratings. By adjusting the device diameter through the wet etching technique, the sensitivity of hydrogen concentration detection is improved by 2.7 times, which provides a good platform for reliable and safe hydrogen sensing and simultaneous temperature monitoring. Ahmad et al. [107] proposed a novel sensor based on back-reflective fiber Bragg grating signal strength assessment by coating palladium nanoparticles on the sensing portion of etched single-mode fiber. It has a dynamic operating range of 0.3–5% at room temperature, fast response and recovery without hysteresis, and palladium nanoparticles are antioxidants.

Fiber optic hydrogen sensors can be used at any temperature without the risk of explosion due to the detection of optical signals only and have a high safety coefficient. In special use scenarios, only the wavelength of the optical fiber needs to be adjusted appropriately to achieve long-distance measurements. However, the structure of a fiber-optic hydrogen sensor is complicated, and the preparation cost is very high, which makes it difficult to be widely used.

## 4. Conclusions

Currently, hydrogen sensors fabricated with palladium metal as the hydrogen-sensitive material are continuously innovating in structure. However, they still face many challenges in future development, such as improving the gas selectivity of the sensor when the hydrogen concentration is below 1% and reducing the interference of other gas molecules on their performance. This paper analyzes in detail the advantages and disadvantages of four types of palladium-based hydrogen sensors: Sensors based on palladium films have high sensitivity and fast response characteristics and are suitable for high-precision detection scenarios, but the cost is relatively high. Palladium alloy sensors perform outstandingly in terms of stability and anti-interference ability, are suitable for complex environment applications, and have relatively low manufacturing costs. Palladium nanoparticle-modified sensors, with their unique nano-effect, can effectively detect low-concentration hydrogen. However, the complex process leads to an increase in cost. Palladium composite material sensors perform well in comprehensive performance and have a medium cost. Meanwhile, the MEMS process has significant advantages in the preparation of PD-based resistive hydrogen sensors. This process can achieve the miniaturization of sensors, significantly reduce the size of devices, and facilitate integration and portable applications. It can enhance manufacturing accuracy, precisely control the structure and distribution of palladium materials, and optimize the performance of sensors. It can also achieve batch production, reduce the unit manufacturing cost, and improve production efficiency. Through the design of micro-nano structures, the contact area between palladium and hydrogen is increased, enhancing the sensitivity and response speed of the sensor.

Finally, by comprehensively comparing various types of hydrogen sensors, it can be known that in large-scale industrial detection scenarios that pursue high cost performance, palladium alloy sensors have the best cost-effectiveness because they balance performance and cost. In fields with extremely high precision requirements, such as scientific research experiments and semiconductor manufacturing, although palladium film sensors are costly, their outstanding detection performance still makes them the preferred choice. The emerging hydrogen economy continues to drive technological innovation in sensors. In the future, palladium-based hydrogen sensors need to delve deeply into structural optimization and material innovation to fully meet the diverse demands of hydrogen safety applications.

## Data Availability

The data presented in this study are available on request from the corresponding author.
